# A Plea for Diversity in Eating Disorders Research

**DOI:** 10.3389/fpsyt.2022.820043

**Published:** 2022-02-18

**Authors:** Georg Halbeisen, Gerrit Brandt, Georgios Paslakis

**Affiliations:** University Clinic for Psychosomatic Medicine and Psychotherapy, Medical Faculty, Campus East-Westphalia, Ruhr-University Bochum, Luebbecke, Germany

**Keywords:** eating disorders, social diversity, gender bias, ethnic bias, sexual minority, medical education

## Abstract

Eating disorders (EDs) are often stereotyped as affecting the SWAG, that is, as affecting mostly skinny, White, affluent girls. Over the last decade, however, significant progress has been made toward increasing diversity in ED research. There is consensus that EDs affect individuals of all genders, ages, sexual orientations, ethnic, and socio-economic backgrounds, with recent studies exploring social determinants of ED etiology, ED presentation, and developing diversity-affirming ED assessments. This article provides a brief summary of current developments related to diversity as a research theme, and proposes different perspectives toward further improving diversity in ED research. Specifically, we argue for exploring the role of diversity in ED treatment settings and outcomes, for pursuing diversity-oriented research pro-actively rather than as a reaction to issues of under-representation, and for integrating diversity across different areas of medical education and trainings in psychotherapy. Limitations with respect to the paucity of research, and the link between diversity as a research theme and ED-related workforce diversity are discussed.

## Introduction

Eating disorders (EDs) are of increasing public health concern (1). Characterized by body image concerns, abnormal eating, and weight-control behaviors (2), about 1.69% of the global population suffer from an ED such as Anorexia Nervosa, Bulimia Nervosa, or Binge Eating Disorder during their lifetime (3, 4). EDs pose one of the highest mortality risks among mental disorders (5) and are associated with adverse physical and mental health outcomes across multiple domains of functioning (6). Untreated ED-related healthcare and economic costs are substantial (7), emphasizing the importance of early detection and tailoring treatments toward ED patients' needs.

Counter to the widespread perception that EDs affect mostly White, adolescent girls from wealthy, industrialized countries (7, 8)—a stereotype colloquially labeled SWAG: skinny, White, affluent girls (9, 10)—epidemiological data show that individuals of all genders, sexual orientations, ages, ethnicities, and socio-economic status suffer from EDs (3, 4). In fact, recent years witnessed an unprecedented increase in ED incidence among men (11, 12), older adults (13, 14), gender and sexual minority individuals (15), underrepresented ethnic groups (16, 17), and adults with intellectual disabilities (18–20). Although wealthier individuals are more likely to receive ED treatment (9), the existing data suggest that EDs present equally across different socio-economic backgrounds (21). The changing demographics of the ED population point to a critical role of diversity in ED etiology, broadly defined in terms of any social or individual identity features that lead to the perception of differences between people (22), with possible implications for ED presentation and assessment, and the necessity for adjustments according to diverse treatment needs. At the same time, diversity issues such as gender, age, and ethnicity only recently rose to prominence as a subject of investigation in ED research.

**Figure 1:**
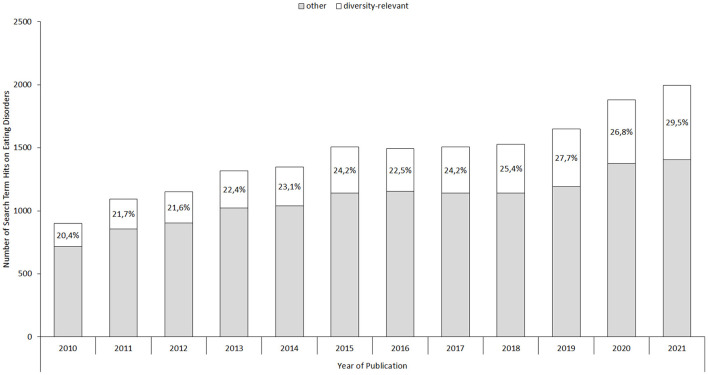
Illustration of the number of yearly total ED publications and potentially diversity-relevant ED publications. Percentages indicate the proportion of diversity-relevant among total yearly ED publications.

Figure 1 illustrates the diversity-related publication trend between 2010 and late 2021. Specifically, we queried PubMed (www.pubmed.gov) on November 11th, 2021, for studies published since 2010 on eating disorders (i.e., eating disorder, anorexia nervosa, bulimia nervosa, binge eating, OSFED, or EDNOS), as mentioned in their title or abstract, which revealed 17,374 hits. In conjunction with diversity-related search terms (divers^*^, trans^*^, gay, lesbian, bisexual, ethnic, racial, minority, male, men, gender, elderly, older, and disability), a subgroup of 4,282 hits were identified. Stratified by publication year, the raw figures suggest up to a 1.5-times increase in the proportion of diversity-relevant among the total number of ED publications within the last decade (20.4 vs. 29.5%), with absolute publication numbers showing upwards of a three-fold increase (from 184 to 590 publications). These raw figures likely overestimate the number of publications specifically addressing diversity-issues in ED research due to the broadly defined search terms. Nevertheless, the increase in yearly search term hits shows an increase in referencing diversity-related themes, suggesting that diversity features are increasingly recognized in ED research.

A potential reason for the historic inattention to diversity features in ED research and the need for its continued advancement is the lack of diversity among the ED research and professional communities. A recent survey attests that the global ED workforce is mainly comprised of White, cis-gendered women (23), with survey respondents recognizing high costs of education, limited social encouragement, and the lack of “leadership”—positions of power within the field are held mainly by White men (24–26)—as barriers to increasing workforce diversity. Improving workforce diversity will encourage individuals from underrepresented groups to seek treatment and promote patient-centered care, ultimately leading to the inclusion of their experiences and perspectives in the scientific discussion. Thus, as a caveat to further discussing the increasing interest in diversity-related themes, one may be wary that positive effects of this trend might be thwarted if left unaccompanied by overarching changes aiming at improving diversity representation among proponents of the field. At the same time, positive effects of improving workforce diversity could be undermined if solely relying on expecting diverse professionals to promote diversity in ED research (27). This suggests that independently discussing and advancing diversity as a research theme—as one of several levels of the field's diversity challenge—is warranted.

This article therefore presents a non-exhaustive summary of significant developments made with respect to investigating the role of diversity in ED etiology, presentation, and assessment. We present our perspective on advancing diversity as a research theme further, suggesting that future ED studies may focus on exploring the role of diversity in ED treatment settings and outcomes, anticipating diversity challenges, and investigating diversity perspectives across different areas of medical education and psychotherapeutic trainings.

## Current Advances

### Diversity as a Risk Factor in Eating Disorder Etiology

Higher rates of ED prevalence found among Asian-American men, Black women, transgender, and other sexual minority individuals compared to White, cis-gender women (15, 16, 28, 29) suggest that diversity-related features may be prominently associated to ED development. As a result, interest in understanding ED etiology as it links to diversity has increased. Two prominent models may be discussed that could link diversity to EDs: The socio-cultural model (30), which focusses on conformity with (sub-)cultural appearance ideals and peer-pressure as contributing factors to EDs, and the minority stress model (31), which posits that increased levels of stigma-related stress associated to being part of a social minority group would lead to higher burden of EDs. In a similar vein, the stress of adapting to appearance ideals of a majority group, i.e., acculturative stress, has been associated with increased ED symptoms in ethnic minority women (32). Convertino et al. (33) recently compared and integrated socio-cultural and stress models for addressing EDs in sexual minority individuals. Consistent with the models' central predictions, the authors reported that involvement within a sexual minority community increases appearance and body-ideal pressures, while stress-related experiences of heterosexist discrimination predict body-related dissatisfaction. However, it remains to be further empirically explored whether these findings can be generalized toward increased prevalence rates of EDs among ethnic minorities, in different age groups, or toward men, who represent a minority in the realm of EDs in contrast to other areas of clinical research (34). Nevertheless, these and similar findings highlight the complex ways in which group and gender identities may influence the development of EDs.

Burke et al. (35) recently advocated for extending diversity-related etiological models to account for interaction effects of occupying multiple diversity phenotypes in ED development. That is, the authors point to preliminary evidence showing that individuals with intersecting identities (e.g., ethnic *and* gender minority status) have an increased risk for EDs that is greater than the risk of each feature separately (36). Though the additional layer of complexity in investigating diversity in ED research poses methodological and sampling challenges (35), it also provides the narrative that focusing on single diversity-related features may create blind spots for recognizing certain (sub)groups with increased vulnerabilities.

### Diversity in Eating Disorder Presentation

Significant progress has also been made toward identifying links between diversity and ED presentation. For example, men with EDs are often concerned with achieving a muscular, well-defined body ideal rather than thinness, which suggests different disordered eating patterns and weight-control behaviors compared to women (37–39), including anabolic steroid use associated with muscle dysmorphia (40) and different patterns in emotion regulation (41). Among transgender youth and adolescents with ED, suicidal ideations and suicide attempts are significantly elevated compared to cis-gender youth with EDs (42). Moreover, restricting feeding patterns in transgender youth may serve to delay the development of secondary sex characteristics and may thus additionally function to cope with gender-related distress (15). Finally, preliminary findings suggest that older individuals could be more adept at masking ED symptoms (43), although their symptoms may not fundamentally differ from younger populations (44).

It remains to be clarified whether and to what extend variations in ED presentation directly reflect diversity-related socio-cultural influences. The current evidence points toward the influence of gender and ethnic community norms on symptom reporting (45), differences in self-perception of symptom presence or severity (46), and help-seeking behavior (47). Final conclusions may be premature, as such variations in ED presentation may also reflect unequal access to treatment and health care services across diverse groups (48) or clinician bias (49). Others note that the specificity of diversity effects in ED presentation across diagnostic subgroups (e.g., Anorexia Nervosa, Bulimia Nervosa, Binge Eating Disorder) needs further exploration, too (12). The need for diversity-affirming ED diagnostic criteria and assessments is nonetheless warranted.

### Updates in Diagnostic Criteria

Updates in diagnostic criteria mirror an increased recognition of the role of diversity across the ED patient population (44). Specifically, the fifth edition of the Diagnostic and Statistical Manual of Mental Disorders (2) was the first to include Binge Eating Disorder as a specific diagnosis, which is an ED more commonly found in men (3), older individuals (50), and more prevalent in ethnic minority individuals (51). Amenorrhea was removed as a criterion for anorexia nervosa, which formerly excluded men, post-menopausal women as well as women receiving hormonal treatments (52). Although the discussion about the socio-cultural appropriateness of specific criteria continues (45), including the use of the body mass index as discriminating against Black and Asian individuals (53, 54), the existing updates in diagnostic criteria represent an important step toward equalizing treatment access irrespective of diverse backgrounds.

Further attention is directed toward the diversity-related appropriateness of screening and diagnostic tools, which several authors recognize were predominantly developed and validated among White women (39, 55). Recent diversity-affirming ED research, therefore, includes norming and reformulation efforts for standardized ED assessments such as the Eating Disorder Inventory and the Eating Disorder Examination-Questionnaire for heterosexual men (56), gay men (57), transgender persons (58, 59), and Black patients (60). Although some of these efforts have revealed a convergence of questionnaire structures between diverse samples (61, 62), others have shown that identical questionnaires may measure dissimilar constructs in different diverse groups (63), emphasizing the necessity for validation across groups.

## Perspectives for Future Diversity-Related ED Research

Identifying diversity-related risk factors, differences in ED presentation and the development of diversity-affirming diagnostic criteria and tools are vital to early detect EDs and address existing disparities in treatment access. At the same time, diversity as a research theme should not be limited to exploring its role in equalizing detection and treatment access but should be further extended to investigating the role of diversity in treatment settings (with potential inferences to treatment outcomes), anticipating future research challenges in ED treatment, and investigating diversity representation in professional education and training.

### Diversity in Treatment Settings

Reports on treatment experiences of individuals from minority and/or marginalized groups indicate the need to improve diversity-related infrastructure and content in treatment settings. A prominent line of examples concerning diversity-related treatment experiences was recently summarized in a qualitative systematic review by our group (64). We reported that men with EDs face unique challenges during treatment, often struggling to feel understood by therapists (65), feeling unwanted in the treatment environment (66), experiencing marginalization in otherwise women-dominated facilities (67), or feeling that men-specific experiences and concerns are not taken into account (66). Similar reports among transgender youth and adolescents with EDs suggest that gender-related concerns are not or inadequately addressed during treatment (15, 29, 68, 69). One may suspect that the overrepresentation of White, cis-gender women in ED research may have inadvertently facilitated the development of treatment settings geared toward that population (70), again, displaying the need to tailor ED treatments according to the concerns and expectations of diverse groups. Rodgers et al. (16) summarize first instances of tailored ED treatments for individuals from underrepresented ethnic groups. For example, based on focus-group feedback, Shea et al. (71) suggest adaptations for cognitive behavioral therapy (CBT) for EDs with Mexican-American women in terms of addressing cultural beliefs regarding ED etiology, social meanings of food and eating, and cultural preferences in help-seeking behavior. Similarly, Accurso et al. (72) recently published a protocol for adapting family-based treatment toward the needs of socio-economically disadvantaged and racially diverse adolescents. However, this avenue remains largely unexplored, as specific treatments targeting EDs in men, sexual minority individuals, or older adults are still lacking (70, 73, 74).

The impact of negative treatment experiences upon treatment outcomes in diverse groups is another area of needed research. Unlike in most other areas of clinical research (34), women constitute the majority in samples of randomized controlled trials that examine ED treatment efficacy, thus limiting conclusions about treatment outcomes in other groups. For illustrative purposes, we calculated the average sample composition of more than 300 studies summarized in the current German treatment guidelines (75), which recommend psychotherapy, primarily CBT, as first-choice intervention for adults with EDs. We found that women represented 90%, 95%, and more than 99% of total samples in studies on binge eating disorder, anorexia nervosa, and bulimia nervosa, respectively. As the under-representation of diverse groups in outcome-related ED trials may have inadvertently facilitated the development of more effective treatments for cis-gender women, enhancing diversity representation in clinical ED trials is vital for ensuring optimal health care delivery and identifying areas in need of further improvement (76). First examples of gender-based comparisons suggest equality among treatment outcomes (77–81), but there is still a dearth of systematic investigations concerning gender and other diversity-related differences in outcome-related research.

Initial steps toward increasing diversity among ED trial populations may reside in identifying barriers to ED study participation. Using a mixture of reviews, field testing, and expert and patient interviews, Clark et al. (82) recently identified a lack of trust among patients from underrepresented groups as well as a lack of awareness of the importance of diversity among physicians and investigators as barriers to the execution of trials in diverse populations in general. Suggested solutions included involving patients in study design, offering adequate compensation, providing structured support to referring physicians, and training principal investigators, staff, and coordinators in cultural competency. Similar approaches could be used to both identify barriers to ED trial participation and develop ED-specific solutions as well.

Another key obstacle to advancing our understanding of the role of diversity in ED treatment settings and outcomes are reporting practices in clinical trials, which rarely address sample compositions beyond average age and sex or include diversity-oriented subgroup comparisons. Moreover, there is no consensus on how diversity and which aspects of it should be measured, rendering direct comparisons difficult. Therefore, we advocate for developing diversity-oriented reporting standards for clinical trials in ED research. As diversity issues may not be the focus of most future clinical ED trials, reporting standards could facilitate diversity-oriented systematic reviews and meta-analyses. Tools for guideline development are readily available (83), with initial examples demonstrating the feasibility of similar endeavors (84). Given the inevitably lower number of cases with the increasing complexity of subgroup comparisons, establishing reporting standards may also help to address methodological concerns related to the investigation of diversity effects (35, 85).

### Anticipating Diversity Challenges

Differences in treatment access and outcomes represent a strong imperative for investigating existing inequalities associated with the affiliation to one or more diverse groups. To prevent further disparities, however, future research may also need to anticipate diversity challenges based on current developments, supporting the transition from a problem-focused toward an opportunity-focused approach (35). For example, we would argue that socio-economic and educational diversity will become increasingly relevant in ED etiology and treatment. Although at present there are no clear links between diversity in terms of socio-economic background and the prevalence of EDs (21), level of education, and other factors associated with socio-economic status have been linked to food insecurity during the Covid-19 pandemic (86). Food insecurity, in turn, has been repeatedly linked to ED pathology (87), suggesting that future ED populations may disproportionally stem from lower-income and lower-education households. Indeed, although affected at similar rates, past research suggests that individuals from lower socio-economic backgrounds show increased health impairments due to EDs (88). Thus, it may be necessary to adjust treatment approaches toward anticipating differences in literacy and other education-related aspects of the patients and provide patients from less privileged backgrounds with additional access options to disorder-specific treatments. Although these implications are not entirely different from those proposed for adjusting psychotherapy for patients with intellectual disabilities (89) or for an aging patient population (90, 91) -which is another challenge ED research might soon face -the effort of their implementation will likely outweigh the social and economic costs of additional social disparities in ED treatment.

Admittedly, systematic projections about future diversity challenges in ED research remain limited by contemporary diversity conceptualizations, which distinguish within the aspects of ED etiology, presentation, and treatment access and outcomes by categorizing individuals based on sex, gender, age, ethnicity, or sexual orientation. Not only is it questionable whether these categories are representative of ED populations on a global scale (3), but grouping EDs based on social categories inevitably incurs the risk of an ecological fallacy, i.e., of inferring individual-level causation from group-level correlation. In other words, uncertainty remains about the causal mechanism(s) linking diversity to ED treatment challenges, as only a limited number of universal approaches, such as the minority stress model (31), have yet been proposed. Therefore, anticipating diversity challenges may require further research on individual-level factors linking diversity to ED outcomes and experiences.

### Diversity in ED-Related Clinical Education

The diverse treatment needs of individuals with EDs are challenging to address without incorporating diversity aspects in clinical trainings (physicians, psychologists, psychotherapists). Numerous examples provide evidence for a lack of awareness regarding diversity issues among medical personnel, such that individuals of sexual minorities report a lack of trust in disclosing their sexual preferences (92, 93). Likewise, medical professionals report uncertainty in dealing with diversity issues (71) and feel that these issues were not adequately addressed as part of their education or training (94). Based on these and similar findings, there have been numerous calls to systematically incorporate diversity-related perspectives in medical education (95–97), including the representation of diversity in medical textbooks (98).

To the best of our knowledge, there are no diversity-oriented medical trainings that focus specifically on ED interventions or training. Some contemporary examples of diversity-oriented medical trainings have been evaluated at mostly North-American universities and institutions, although there are only a few examples of extensive curricula (99, 100). Furthermore, diversity trainings of the kind are often offered separately from general education, usually geared toward specific minority groups, and offered voluntarily, thus bearing the risk that only a limited number of students (e.g., those with prior knowledge and interest in the subject, and the willingness to question their positions) will participate. The voluntary nature of these classes could reinforce the perception of diversity issues as an afterthought rather than an essential component in medical education, which, in turn, may potentially affect the provision of care to patients.

While existing training may serve as a template for developing diversity-oriented ED curricula, a holistic approach sensitizing for diversity throughout professional education and training may be preferable. Such an approach not only requires building an extensive, evidence-based knowledge base on the critical role of diversity in ED presentation and treatment but may involve engaging, sensitizing, motivating, and diversifying both student bodies, teaching staff, and faculty administrations.

## Discussion

Similar to how “swag” lost its popularity as a decade-old teenage slang, the acronym has become decreasingly less relevant for describing patient populations in ED research. There have been numerous advances toward understanding the role of diversity in ED etiology and ED presentation, with updated diagnostic criteria and the development of diversity-affirming diagnostic tools to target the existing disparities in treatment access.

Complementing existing efforts, we proposed that future ED research would need to focus on addressing the role of diversity in treatment experiences and disparities in outcomes, which may require adjusting recruitment, assessment, and reporting practices in clinical trials. Moreover, anticipating future developments that could pose diversity challenges and incorporating diversity-related issues systematically in ED-related clinical training are asked for, though tackling these issues may require considerable effort to extend theoretical conceptualizations of diversity and develop interventional approaches.

Of course, our perspective is itself limited by the general paucity of evidence, a majority of which originates from US-American and European contexts and authors, with research participants often recruited from populations that already have access to (private) healthcare treatment. Therefore, it would seem appropriate to also incorporate diverse, i.e., global research perspectives into the study of ED diversity, as well as perspectives of currently underserved groups.

As alluded to in the introduction, the potential benefits of advancing ED diversity as a research theme further depend upon improving workforce diversity. For example, study participation could be hindered if patients feel unwelcomed in research environments due to low diversity representation. Similarly, incorporating diversity within medical trainings could be ineffective at best and hypocritical at worst without addressing access barriers to medical education and training for individuals from underrepresented backgrounds. Advancing ED diversity thus remains a multi-level challenge that is aided but not solved by increasing research diversity.

## Data Availability Statement

The original contributions presented in the study are included in the article/supplementary material, further inquiries can be directed to the corresponding author.

## Author Contributions

GP and GH conceived the topic and proposition of this manuscript. GH wrote the first draft. All authors contributed to manuscript revision, read, and approved the submitted version.

## Funding

We acknowledged the support by DFG Open Access Publication Funds of the Ruhr-Universität Bochum.

## Conflict of Interest

The authors declare that the research was conducted in the absence of any commercial or financial relationships that could be construed as a potential conflict of interest.

## Publisher's Note

All claims expressed in this article are solely those of the authors and do not necessarily represent those of their affiliated organizations, or those of the publisher, the editors and the reviewers. Any product that may be evaluated in this article, or claim that may be made by its manufacturer, is not guaranteed or endorsed by the publisher.

## References

[1] Treasure J Duarte TA Schmidt U. Eating disorders. Lancet. (2020) 395:899–911. 10.1016/S0140-6736(20)30059-3 32171414

[2] American Psychiatric Association. Diagnostic and Statistical Manual of Mental Disorders. 5th ed. Washington, DC:? American Psychiatric Publishing (2013).

[3] Qian J Wu Y Liu F Zhu Y Jin H Zhang H. An update on the prevalence of eating disorders in the general population: a systematic review and meta-analysis. Eat Weight Disord. (2021) 1–14. 10.1007/s40519-021-01162-z 33834377 PMC8933366

[4] Santomauro DF Melen S Mitchison D Vos T Whiteford H Ferrari AJ. The hidden burden of eating disorders: an extension of estimates from the Global Burden of Disease Study 2019. Lancet Psychiatry. (2021) 8:320–328. 10.1016/S2215-0366(21)00040-7 33675688 PMC7973414

[5] Chesney E Goodwin GM Fazel S. Risks of all-cause and suicide mortality in mental disorders: a meta-review. World Psychiatry. (2014) 13:153–60. 10.1002/wps.20128 24890068 PMC4102288

[6] Udo T Grilo CM. Psychiatric and medical correlates of DSM-5 eating disorders in a nationally representative sample of adults in the United States. Int J Eat Disord. (2019) 52:42–50. 10.1002/eat.23004 30756422

[7] Douglas V Balas B Gordon K. Facial femininity and perceptions of eating disorders: a reverse-correlation study. PLoS ONE. (2021) 16:e0255766. 10.1371/journal.pone.0255766 34358270 PMC8345843

[8] Gordon KH Perez M Joiner TE. The impact of racial stereotypes on eating disorder recognition. Int J Eat Disord. (2002) 32:219–224. 10.1002/eat.10070 12210665

[9] Sonneville KR Lipson SK. Disparities in eating disorder diagnosis and treatment according to weight status, race/ethnicity, socioeconomic background, and sex among college students. Int J Eat Disord. (2018) 51:518–26. 10.1002/eat.22846 29500865

[10] Pike KM Dunne PE Addai E. Expanding the boundaries: reconfiguring the demographics of the “typical” eating disordered patient. Curr Psychiatry Rep. (2013) 15:1–8. 10.1007/s11920-013-0411-2 24122512

[11] Madden S Morris A Zurynski YA Kohn M Elliot EJ. Burden of eating disorders in 5–13-year-old children in Australia. Med J Aust. (2009) 190:410–4. 10.5694/j.1326-5377.2009.tb02487.x 19374611

[12] Sangha S Oliffe JL Kelly MT McCuaig F. Eating disorders in males: how primary care providers can improve recognition, diagnosis, and treatment. Am J Mens Health. (2019) 13:1–12. 10.1177/1557988319857424 31184292 PMC6560809

[13] Mangweth-Matzek B Kummer KK Pope HG. Eating disorder symptoms in middle-aged and older men. Int J Eat Disord. (2016) 49:953–7. 10.1002/eat.22550 27173753

[14] Mangweth-Matzek B Hoek HW Pope HG. Pathological eating and body dissatisfaction in middle-aged and older women. Curr Opin Psychiatry. (2014) 27:431–5. 10.1097/YCO.0000000000000102 25247455

[15] Coelho JS Suen J Clark BA Marshall SK Geller J Lam PY. Eating disorder diagnoses and symptom presentation in transgender youth: a scoping review. Curr Psychiatry Rep. (2019) 21:1–10. 10.1007/s11920-019-1097-x 31617014

[16] Rodgers RF Berry R Franko DL. Eating disorders in ethnic minorities: an update. Curr Psychiatry Rep. (2018) 20:1–11. 10.1007/s11920-018-0938-3 30155577

[17] Cheng ZH Perko VL Fuller-Marashi L Gau JM Stice E. Ethnic differences in eating disorder prevalence, risk factors, and predictive effects of risk factors among young women. Eat Behav. (2019) 32:23–30. 10.1016/j.eatbeh.2018.11.004 30529736 PMC6382562

[18] Gravestock S. Eating disorders in adults with intellectual disability. J Intellect Disabil Res. (2000) 44:625–37. 10.1046/j.1365-2788.2000.00308.x 11115017

[19] Hove O. Prevalence of eating disorders in adults with mental retardation living in the community. Am J Ment Retard. (2004) 109:501–06. 10.1352/0895-8017(2004)109<501:POEDIA>2.0.CO;2 15471515

[20] Westwood H Tchanturia K. Autism spectrum disorder in anorexia nervosa: an updated literature review. Curr Psychiatry Rep. (2017) 19:41. 10.1007/s11920-017-0791-9 28540593 PMC5443871

[21] Huryk KM Drury CR Loeb KL. Diseases of affluence? A systematic review of the literature on socioeconomic diversity in eating disorders. Eat Behav. (2021) 43:101548. 10.1016/j.eatbeh.2021.101548 34425457

[22] Roberge MÉ van Dick R. Recognizing the benefits of diversity: when and how does diversity increase group performance? Hum Resour Manag Rev. (2010) 20:295–308. 10.1016/j.hrmr.2009.09.002

[23] Jennings Mathis K Anaya C Rambur B Bodell LP Graham AK Forney KJ. Workforce diversity in eating disorders: a multi-methods study. West J Nurs Res. (2020) 42:1068–77. 10.1177/0193945920912396 32266857 PMC7541546

[24] Wingard D Trejo JA Gudea M Goodman S Reznik V. Faculty equity, diversity, culture and climate change in academic medicine: a longitudinal study. J Natl Med Assoc. (2019) 111:46–53. 10.1016/j.jnma.2018.05.004 30129483

[25] Filardo G Graca B Da Sass DM Pollock BD Smith EB Martinez MAM. Trends and comparison of female first authorship in high impact medical journals: observational study (1994-2014). BMJ. (2016) 352:1–8. 10.1136/bmj.i847 26935100 PMC4775869

[26] Pinho-Gomes AC Vassallo A Thompson K Womersley K Norton R Woodward M. Representation of women among editors in chief of leading medical journals. JAMA Netw Open. (2021) 4:2123026. 10.1001/jamanetworkopen.2021.23026 34495341 PMC8427369

[27] Laver KE Prichard IJ Cations M Osenk I Govin K Coveney JD. A systematic review of interventions to support the careers of women in academic medicine and other disciplines. BMJ Open. (2018) 8:20380. 10.1136/bmjopen-2017-020380 29572397 PMC5875640

[28] Goode RW Cowell MM Mazzeo SE Cooper-Lewter C Forte A Olayia OI. Binge eating and binge-eating disorder in black women: a systematic review. Int J Eat Disord. (2020) 53:491–507. 10.1002/eat.23217 31922293 PMC8010989

[29] Nagata JM Ganson KT Austin SB. Emerging trends in eating disorders among sexual and gender minorities. Curr Opin Psychiatry. (2020) 33:562–7. 10.1097/YCO.0000000000000645 32858597 PMC8060208

[30] Hazzard VM Schaefer LM Schaumberg K Bardone-Cone AM Frederick DA Klump KL. Testing the tripartite influence model among heterosexual, bisexual, and lesbian women. Body Image. (2019) 30:145–9. 10.1016/j.bodyim.2019.07.001 31323438 PMC6703947

[31] Meyer IH. Prejudice, social stress, and mental health in lesbian, gay, and bisexual populations: conceptual issues and research evidence. Psychol Bull. (2003) 129:674–97. 10.1037/0033-2909.129.5.674 12956539 PMC2072932

[32] Kroon Van Diest AM Tartakovsky M Stachon C Pettit JW Perez M. The relationship between acculturative stress and eating disorder symptoms: is it unique from general life stress? J Behav Med. (2014) 37:445–57. 10.1007/s10865-013-9498-5 23456250

[33] Convertino AD Helm JL Pennesi JL Gonzales M Blashill AJ. Integrating minority stress theory and the tripartite influence model: a model of eating disordered behavior in sexual minority young adults. Appetite. (2021) 163:105204. 10.1016/j.appet.2021.105204 33741450

[34] Feldman S Ammar W Lo K Trepman E Van Zuylen M Etzioni O. Quantifying sex bias in clinical studies at scale with automated data extraction. JAMA Netw Open. (2019) 2:e196700. 10.1001/jamanetworkopen.2019.6700 31268541 PMC6613296

[35] Burke NL Schaefer LM Hazzard VM Rodgers RF. Where identities converge: the importance of intersectionality in eating disorders research. Int J Eat Disord. (2020) 53:1605–9. 10.1002/eat.23371 32856342 PMC7722117

[36] Beccia AL Baek J Jesdale WM Austin SB Forrester S Curtin C. Risk of disordered eating at the intersection of gender and racial/ethnic identity among U.S. high school students. Eat Behav. (2019) 34:101299. 10.1016/j.eatbeh.2019.05.002 31153023

[37] Nagata JM Ganson KT Murray SB. Eating disorders in adolescent boys and young men: an update. Curr Opin Pediatr. (2020) 32:476–81. 10.1097/MOP.0000000000000911 32520822 PMC7867380

[38] Núñez-Navarro A Agüera Z Krug I Jiménez-Murcia S Sánchez I Araguz N. Do men with eating disorders differ from women in clinics, psychopathology and personality? Eur Eat Disord Rev. (2012) 20:23–31. 10.1002/erv.1146 21823213

[39] Murray SB Nagata JM Griffiths S Calzo JP Brown TA Mitchison D. The enigma of male eating disorders: a critical review and synthesis. Clin Psychol Rev. (2017) 57:1–11. 10.1016/j.cpr.2017.08.001 28800416

[40] Badenes-Ribera L Rubio-Aparicio M Sánchez-Meca J Fabris MA Longobardi C. The association between muscle dysmorphia and eating disorder symptomatology: a systematic review and meta-analysis. J Behav Addict. (2019) 8:351–71. 10.1556/2006.8.2019.44 31505966 PMC7044626

[41] Agüera Z Paslakis G Munguía L Sánchez I Granero R Sánchez-González J. Gender-related patterns of emotion regulation among patients with eating disorders. J Clin Med. (2019) 8:161. 10.3390/jcm8020161 30717125 PMC6406611

[42] Duffy ME Henkel KE Joiner TE. Prevalence of self-injurious thoughts and behaviors in transgender individuals with eating disorders: a national study. J Adolesc Heal. (2019) 64:461–6. 10.1016/j.jadohealth.2018.07.016 30314865

[43] Zayed M Garry JP. Geriatric anorexia nervosa. J Am Board Fam Med. (2017) 30:666–9. 10.3122/jabfm.2017.05.170182 28923819

[44] Mulchandani M Shetty N Conrad A Muir P Mah B. Treatment of eating disorders in older people: a systematic review. Syst Rev. (2021) 10:275. 10.1186/s13643-021-01823-1 34696804 PMC8543781

[45] Lee-Winn A Mendelson T Mojtabai R. Racial/ethnic disparities in binge eating: disorder prevalence, symptom presentation, and help-seeking among Asian Americans and non-Latino Whites. Am J Public Health. (2014) 104:1263–5. 10.2105/AJPH.2014.301932 24832409 PMC4056223

[46] MacCaughelty C Wagner R Rufino K. Does being overweight or male increase a patient's risk of not being referred for an eating disorder consult? Int J Eat Disord. (2016) 49:963–6. 10.1002/eat.22556 27203514

[47] Griffiths S Mond JM Li Z Gunatilake S Murray SB Sheffield J. Self-stigma of seeking treatment and being male predict an increased likelihood of having an undiagnosed eating disorder. Int J Eat Disord. (2015) 48:775–8. 10.1002/eat.22413 26052695

[48] Williams DR Lawrence JA Davis BA. Racism and health: evidence and needed research. Annu Rev Public Health. (2019) 40:105–25. 10.1146/annurev-publhealth-040218-043750 30601726 PMC6532402

[49] Becker AE Franko DL Speck A Herzog DB. Ethnicity and differential access to care for eating disorder symptoms. Int J Eat Disord. (2003) 33:205–12. 10.1002/eat.10129 12616587

[50] Smink FRE Van Hoeken D Hoek HW. Epidemiology of eating disorders: incidence, prevalence and mortality rates. Curr Psychiatry Rep. (2012) 14:406–14. 10.1007/s11920-012-0282-y 22644309 PMC3409365

[51] Lydecker JA Grilo CM. Different yet similar: examining race and ethnicity in treatment-seeking adults with binge eating disorder. J Consult Clin Psychol. (2016) 84:88–94. 10.1037/ccp0000048 26348841 PMC4695250

[52] Attia E Roberto CA. Should amenorrhea be a diagnostic criterion for anorexia nervosa? Int J Eat Disord. (2009) 42:581–9. 10.1002/eat.20720 19621464

[53] Fontaine KR Redden DT Wang C Westfall AO Allison DB. Years of life lost due to obesity. J Am Med Assoc. (2003) 289:187–93. 10.1001/jama.289.2.187 12517229

[54] Hood K Ashcraft J Watts K Hong S Choi W Heymsfield SB. Allometric scaling of weight to height and resulting body mass index thresholds in two Asian populations. Nutr Diabetes. (2019) 9:1–7. 10.1038/s41387-018-0068-3 30683839 PMC6347591

[55] Franko DL. Race, ethnicity, and eating disorders: considerations for DSM-V. Int J Eat Disord. (2007) 40:S31–4. 10.1002/eat.20455 17879288

[56] Smith KE Mason TB Murray SB Griffiths S Leonard RC Wetterneck CT. Male clinical norms and sex differences on the eating disorder inventory (EDI) and eating disorder examination questionnaire (EDE-Q). Int J Eat Disord. (2017) 50:769–75. 10.1002/eat.22716 28436086 PMC5741972

[57] Nagata JM Capriotti MR Murray SB Compte EJ Griffiths S Bibbins-Domingo K. Community norms for the eating disorder examination questionnaire among cisgender gay men. Eur Eat Disord Rev. (2020) 28:92–101. 10.1002/erv.2708 31793119 PMC7275693

[58] Duffy ME Calzo JP Lopez E Silverstein S Joiner TE Gordon AR. Measurement and construct validity of the eating disorder examination questionnaire short form in a transgender and gender diverse community sample. Psychol Assess. (2021) 33:459–63. 10.1037/pas0000996 33646808 PMC8756775

[59] Nagata JM Murray SB Compte EJ Pak EH Schauer R Flentje A. Community norms for the eating disorder examination questionnaire (EDE-Q) among transgender men and women. Eat Behav. (2020) 37:101381. 10.1016/j.eatbeh.2020.101381 32416588 PMC7447532

[60] Lydecker JA White MA Grilo CM. Black patients with binge-eating disorder: comparison of different assessment methods. Psychol Assess. (2016) 28:1319–24. 10.1037/pas0000246 26569466 PMC4868800

[61] Belon KE McLaughlin EA Smith JE Bryan AD Witkiewitz K Lash DN. Testing the measurement invariance of the eating disorder inventory in nonclinical samples of Hispanic and Caucasian Women. Int J Eat Disord. (2015) 48:262–70. 10.1002/eat.22286 24740890

[62] Serier KN Peterson KP VanderJagt H Sebastian RM Mullins CR Medici J. Factor analytic support for the EDE-Q7 among American Indian/Alaska Native undergraduate women. Eat Weight Disord. (2021) 10.1007/s40519-021-01335-w 34850357

[63] Spivak-Lavi Z Peleg O Tzischinsky O Stein D Latzer Y. Differences in the factor structure of the eating attitude test-26 (Eat-26) in different cultures in Israel: Jews, muslims, and christians. Nutrients. (2021) 13:1899. 10.3390/nu13061899 34073005 PMC8226938

[64] Richardson C Paslakis G. Men's experiences of eating disorder treatment: a qualitative systematic review of men-only studies. J Psychiatr Ment Health Nurs. (2021) 28:237–50. 10.1111/jpm.12670 32608115

[65] Thapliyal P Conti J Bandara RSL Hay P. “It exists”: an exploratory study of treatment experiences in men with eating disorders. Aust Psychol. (2020) 55:534–45. 10.1111/ap.12455

[66] Robinson KJ Mountford VA Sperlinger DJ. Being men with eating disorders: perspectives of male eating disorder service-users. J Health Psychol. (2013) 18:176–86. 10.1177/1359105312440298 22453166

[67] Kinnaird E Norton C Pimblett C Stewart C Tchanturia K. “There's nothing there for guys”. Do men with eating disorders want treatment adaptations? A qualitative study. Eat Weight Disord. (2019) 24:845–52. 10.1007/s40519-019-00770-0 31471886 PMC6751275

[68] Hartman-Munick SM Silverstein S Guss CE Lopez E Calzo JP Gordon AR. Eating disorder screening and treatment experiences in transgender and gender diverse young adults. Eat Behav. (2021) 41:101517. 10.1016/j.eatbeh.2021.101517 33962139 PMC9645530

[69] Thapliyal P Hay P Conti J. Role of gender in the treatment experiences of people with an eating disorder: a metasynthesis. J Eat Disord. (2018) 6:18. 10.1186/s40337-018-0207-1 30123504 PMC6088416

[70] Lavender JM Brown TA Murray SB. Men, muscles, and eating disorders: an overview of traditional and muscularity-oriented disordered eating. Curr Psychiatry Rep. (2017) 19:32. 10.1007/s11920-017-0787-5 28470486 PMC5731454

[71] Shea M Cachelin F Uribe L Striegel RH Thompson D Wilson GT. Cultural adaptation of a cognitive behavior therapy guided self-help program for Mexican American women with binge eating disorders. J Couns Dev. (2012) 90:308–18. 10.1002/j.1556-6676.2012.00039.x 23645969 PMC3640799

[72] Accurso EC Mu KJ Landsverk J Guydish J. Adaptation to family-based treatment for Medicaid-insured youth with anorexia nervosa in publicly-funded settings: protocol for a mixed methods implementation scale-out pilot study. J Eat Disord. (2021) 9:99. 10.1186/s40337-021-00454-0 34389052 PMC8360814

[73] Goldhammer HB Maston ED Keuroghlian AS. Addressing eating disorders and body dissatisfaction in sexual and gender minority youth. Am J Prev Med. (2019) 56:318–22. 10.1016/j.amepre.2018.09.011 30554976

[74] Mangweth-Matzek B Hoek HW. Epidemiology and treatment of eating disorders in men and women of middle and older age. Curr Opin Psychiatry. (2017) 30:446–51. 10.1097/YCO.0000000000000356 28825955 PMC5690315

[75] Herpertz S Fichter M Herpertz-Dahlmann B Hilbert A Tuschen-Caffier B Vocks S. S3-Leitlinie Diagnostik und Behandlung der Essstörungen. Berlin: Springer (2018).

[76] Gray DM Nolan TS Gregory J Joseph JJ. Diversity in clinical trials: an opportunity and imperative for community engagement. Lancet Gastroenterol Hepatol. (2021) 6:605–7. 10.1016/S2468-1253(21)00228-4 34246352

[77] Strobel C Quadflieg N Naab S Voderholzer U Fichter MM. Long-term outcomes in treated males with anorexia nervosa and bulimia nervosa—A prospective, gender-matched study. Int J Eat Disord. (2019) 52:1353–64. 10.1002/eat.23151 31444805

[78] Lydecker JA Gueorguieva R Masheb R White MA Grilo CM. Examining sex as a predictor and moderator of treatment outcomes for binge-eating disorder: analysis of aggregated randomized controlled trials. Int J Eat Disord. (2020) 53:20–30. 10.1002/eat.23167 31497876 PMC6983351

[79] Fernández-Aranda F Krug I Jiménez-Murcia S Granero R Núñez A Penelo E. Male eating disorders and therapy: a controlled pilot study with one year follow-up. J Behav Ther Exp Psychiatry. (2009) 40:479–86. 10.1016/j.jbtep.2009.06.004 19595294

[80] Coelho JS Suen J Marshall S Burns A Geller J Lam PY. Gender differences in symptom presentation and treatment outcome in children and youths with eating disorders. J Eat Disord. (2021) 9:113. 10.1186/s40337-021-00468-8 34526146 PMC8441244

[81] Murray MF Cox SA Henretty JR Haedt-Matt AA. Women of diverse sexual identities admit to eating disorder treatment with differential symptom severity but achieve similar clinical outcomes. Int J Eat Disord. (2021) 54:1652–62. 10.1002/eat.23576 34260102

[82] Clark LT Watkins L Piña IL Elmer M Akinboboye O Gorham M. Increasing diversity in clinical trials: overcoming critical barriers. Curr Probl Cardiol. (2019) 44:148–72. 10.1016/j.cpcardiol.2018.11.002 30545650

[83] Simera I Moher D Hirst A Hoey J Schulz KF Altman DG. Transparent and accurate reporting increases reliability, utility, and impact of your research: reporting guidelines and the EQUATOR network. BMC Med. (2010) 8:24. 10.1186/1741-7015-8-24 20420659 PMC2874506

[84] Heidari S Babor TF De Castro P Tort S Curno M. Sex and gender equity in research: rationale for the SAGER guidelines and recommended use. Res Integr Peer Rev. (2016) 1:2. 10.1186/s41073-016-0007-6 29451543 PMC5793986

[85] Rodgers RF Donovan E Cousineau TM McGowan K Yates K Cook E. Ethnic and racial diversity in eating disorder prevention trials. Eat Disord. (2019) 27:168–82. 10.1080/10640266.2019.1591824 31084423

[86] Paslakis G Dimitropoulos G Katzman DK. A call to action to address COVID-19-induced global food insecurity to prevent hunger, malnutrition, and eating pathology. Nutr Rev. (2021) 79:114–6. 10.1093/nutrit/nuaa069 32651592 PMC7454780

[87] Hazzard VM Loth KA Hooper L Becker CB. Food insecurity and eating disorders: a review of emerging evidence. Curr Psychiatry Rep. (2020) 22:1–9. 10.1007/s11920-020-01200-0 33125614 PMC7596309

[88] Mitchison D Hay P Slewa-Younan S Mond J. The changing demographic profile of eating disorder behaviors in the community. BMC Public Health. (2014) 14:943. 10.1186/1471-2458-14-943 25213544 PMC4246495

[89] Surley L Dagnan D. A review of the frequency and nature of adaptations to cognitive behavioural therapy for adults with Intellectual Disabilities. J Appl Res Intellect Disabil. (2019) 32:219–237. 10.1111/jar.12534 30353630

[90] Laidlaw K Pachana NA. Aging, mental health, and demographic change: challenges for psychotherapists. Prof Psychol Res Pract. (2009) 40:601–8. 10.1037/a0017215

[91] Groenewald E Joska J Araya R. Psychotherapy adaptation in aging populations. In: Global Mental Health and Psychotherapy: Adapting Psychotherapy for Low- and Middle-Income Countries. London: Elsevier. p. 321–40.

[92] Elliott MN Kanouse DE Burkhart Q Abel GA Lyratzopoulos G Beckett MK. Sexual minorities in england have poorer health and worse health care experiences: a national survey. J Gen Intern Med. (2015) 30:9–16. 10.1007/s11606-014-2905-y 25190140 PMC4284269

[93] Antidiskriminierungsstelle des Bundes. Einstellungen gegenüber Lesben, Schwulen und Bisexuellen in Deutschland. (2017). Available online at: https://www.antidiskriminierungsstelle.de/SharedDocs/downloads/DE/publikationen/Umfragen/umfrage_einstellungen_geg_lesb_schwulen_und_bisex_menschen_de.pdf?__blob=publicationFile&v=4

[94] Turner D Driemeyer W Nieder TO Scherbaum N Briken P. Wie viel sex braucht das studium der medizin?-Eine Erhebung des Wissens und Interesses Medizinstudierender zum Thema Sexualmedizin. PPmP Psychother Psychosom Medizinische Psychol. (2014) 64:452–7. 10.1055/s-0034-1389961 25494185

[95] Maduakolam E Madden B Kelley T Cianciolo AT. Beyond diversity: envisioning inclusion in medical education research and practice. Teach Learn Med. (2020) 32:459–65. 10.1080/10401334.2020.1836462 33349086

[96] Diaz T Navarro JR Chen EH. An institutional approach to fostering inclusion and addressing racial bias: implications for diversity in academic medicine. Teach Learn Med. (2020) 32:110–6. 10.1080/10401334.2019.1670665 31566010

[97] Ludwig S Gruber C Ehlers JP Ramspott S. Diversity in medical education. GMS J Med Educ. (2020) 37:1–6. 10.3205/zma001320 32328529 PMC7171352

[98] Kalantari A Alvarez A Chung A Battaglioli N Nwabueze A Cooney R. 228 sex and racial visual representation in emergency medicine textbooks: a call to action to dismantle the hidden curriculum against diversity in medicine. Ann Emerg Med. (2021) 78:S92. 10.1016/j.annemergmed.2021.09.240

[99] Gibson AW Gobillot TA Wang K Conley E Coard W Matsumoto K. A novel curriculum for medical student training in LGBTQ healthcare : a regional pathway experience. J Med Educ Curric Dev. (2020) 7:1–7. 10.1177/2382120520965254 33195802 PMC7594215

[100] Hawala-Druy S Hill MH. Interdisciplinary: cultural competency and culturally congruent education for millennials in health professions. Nurse Educ Today. (2012) 32:772–8. 10.1016/j.nedt.2012.05.002 22677114

